# Humoral and cellular immune responses after influenza vaccination in patients with chronic fatigue syndrome

**DOI:** 10.1186/1471-2172-13-71

**Published:** 2012-12-17

**Authors:** Hetty Prinsen, I Jolanda M de Vries, Ruurd Torensma, Jeanette M Pots, Sasja F Mulder, Carla M L van Herpen, Lammy D Elving, Gijs Bleijenberg, Foekje F Stelma, Hanneke W M van Laarhoven

**Affiliations:** 1 Department of Medical Oncology, Radboud University Nijmegen Medical Centre, Geert Grooteplein Zuid 8, 6525, GA, Nijmegen, the Netherlands; 2 Department of Tumor Immunology, Radboud University Nijmegen Medical Centre, Nijmegen, the Netherlands; 3 Department of General Internal Medicine, Radboud University Nijmegen Medical Centre, Nijmegen, the Netherlands; 4 Expert Centre for Chronic Fatigue, Radboud University Nijmegen Medical Centre, Nijmegen, the Netherlands; 5 Department of Medical Microbiology, Radboud University Nijmegen Medical Centre, Nijmegen, the Netherlands; 6 Department of Medical Oncology, Academic Medical Center University of Amsterdam, Amsterdam, the Netherlands

**Keywords:** Chronic fatigue syndrome, Influenza, Vaccination, Humoral immunity, Cellular immunity

## Abstract

**Background:**

Chronic fatigue syndrome (CFS) is a clinical condition characterized by severe and disabling fatigue that is medically unexplained and lasts longer than 6 months. Although it is possible to effectively treat CFS, the nature of the underlying physiology remains unclear. Various studies have sought evidence for an underlying disturbance in immunity. The aim of this study was to compare the humoral and cellular immune responses upon influenza vaccination in CFS patients and healthy controls.

**Results:**

Identical antibody titers were observed in CFS patients and healthy controls. Patients and controls demonstrated similar seroprotection rates against all three virus-strains of the influenza vaccine, both pre- and post-vaccination. Functional T cell reactivity was observed in both CFS patients and healthy controls. CFS patients showed a non-significant, numerically lower cellular proliferation at baseline compared to controls. Vaccination induced a significant increase in cellular proliferation in CFS patients, but not in healthy controls. Cytokine production and the number of regulatory T cells were comparable in patients and controls.

**Conclusions:**

The humoral and cellular immune responses upon influenza vaccination were comparable in CFS patients and healthy controls. Putative aberrations in immune responses in CFS patients were not evident for immunity towards influenza. Standard seasonal influenza vaccination is thus justified and, when indicated, should be recommended for patients suffering from CFS.

## Background

Chronic fatigue syndrome (CFS) is a clinical condition characterized by severe and disabling fatigue that is medically unexplained and lasts longer than 6 months [1]. The estimated worldwide prevalence of CFS is 0.4-1% [2]. The existing evidence suggests that cognitive behavior therapy, specifically designed for CFS, is an effective treatment option [3-10]. However, although it is possible to effectively treat CFS, the nature of the underlying pathophysiology remains unclear.

Hypotheses explaining the pathophysiological mechanism of CFS include morphological and metabolic alterations in the brain, [11-17] diminished central activation of muscles, [18] altered central nervous system functioning, [2] a neuroendocrine disturbance, [2] or cognitive impairment [2]. Furthermore, the presence of an underlying immunological problem has been suggested as an explanation for CFS [2]. Cytokine dysregulation, decreased natural killer cell functioning, the presence of autoantibodies, and a reduced response of T cells to mitogens and other specific antigens have been reported in CFS [2,19,20]. If immunity is disturbed in CFS patients, they might have an altered response to vaccination. Vaccines, accompanying adjuvants, and silicone breast implants could act in concert in the development of CFS [2,19-21]. Therefore, the aim of this study was to compare the humoral and cellular immune responses upon vaccination, using seasonal influenza vaccination as a model of a vaccination, in CFS patients and healthy controls.

## Results

### Baseline characteristics

Baseline characteristics of the participants are presented in Table 1.

**Table 1:** Baseline characteristics

	**Chronic fatigue syndrome patients (n=20)**	**Healthy controls (n=20)**	***p*-value**
**Gender**			
Male	7 (35)	7 (35)	1.000
Female	13 (65)	13 (65)	
**Age (years)**			
Mean	35.0±10.0	34.4±9.2	0.857
**CIS-fatigue**	48.15±5.48	15.80±3.62	<0.001
**Hemoglobin (mmol/l)**	8.6±0.6	8.4±0.7	0.235
Mean absolute leukocyte count (*10^**9**^**/l)**	7.2±1.9	6.9±1.9	0.654
**Mean neutrophil count (% of total)**	60.2±8.7	59.9±8.7	0.928
**Mean lymphocyte count (% of total)**	29.8±7.6	29.2±7.1	0.797
**Mean monocyte count (% of total)**	5.7±1.5	5.2±1.4	0.332
**Influenza vaccination prior to 2010**			
Yes	5 (25)	5 (25)	1.000
No	15 (75)	15 (75)	

Data are presented as mean ± standard deviation or as absolute numbers with percentages in brackets.

### Humoral immune response

**Figure 1:**
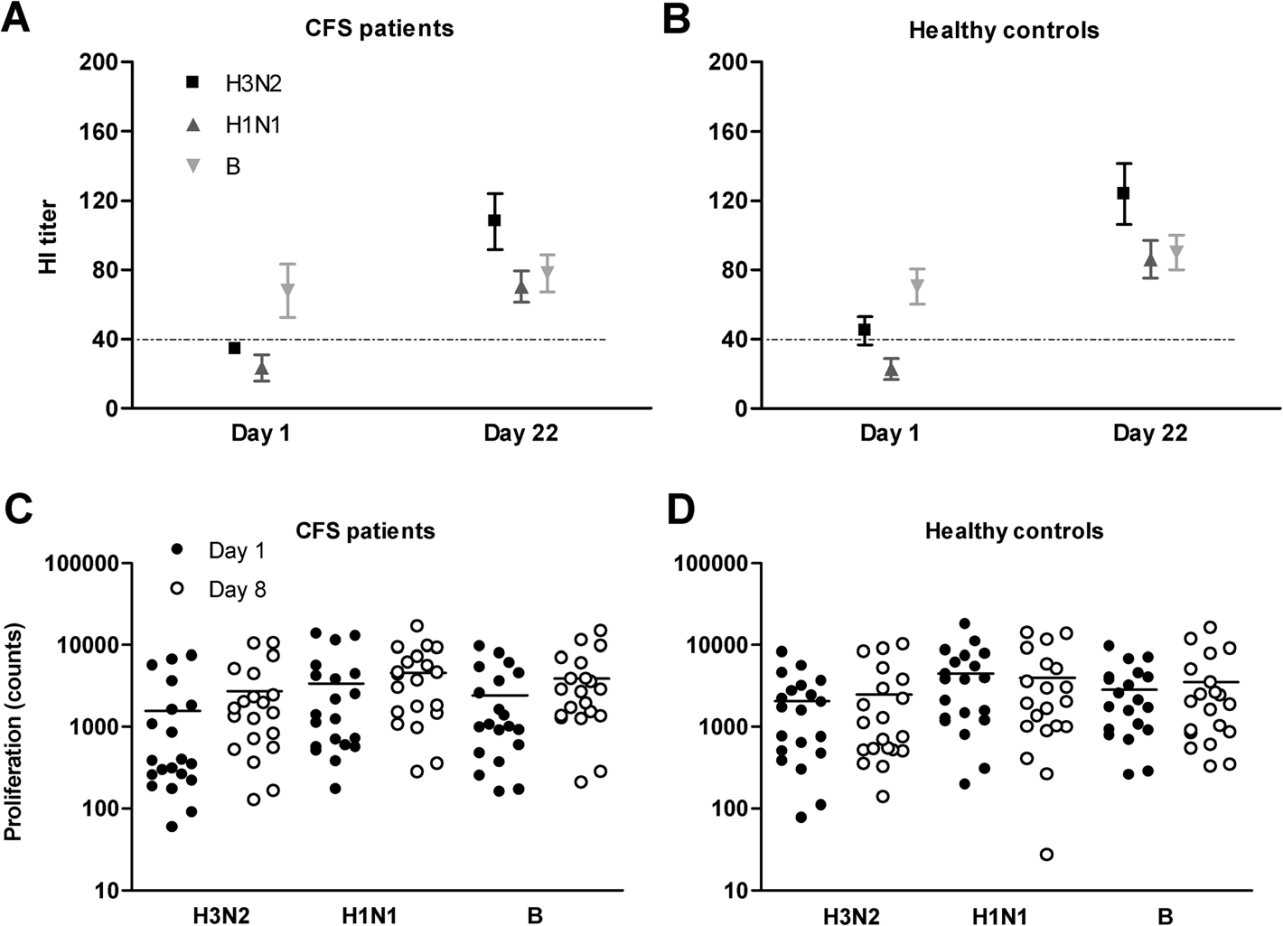
**Hemagglutination inhibition antibody responses before (day 1) and after (day 22) influenza vaccination, and cellular proliferation before (day 1) and after (day 8) influenza vaccination.** Both immune responses are presented for the three influenza strains of the vaccine (H3N2, H1N1, and **B**), in chronic fatigue syndrome patients (**A** and **C**) and healthy controls (**B** and **D**). Antibody titers are presented as mean ± standard error of the mean, the dotted line indicates the protective titer cut-off value. Proliferation counts are presented as absolute numbers and mean (horizontal line) on a logarithmic scale.

Vaccination induced a significant increase in hemagglutination-inhibition (HI) antibody titers for influenza strain H3N2 and H1N1 in the CFS group as well as in the healthy control group (Figure 1A and 1B). For influenza strain B, vaccination induced no significant increase in HI antibody titers, neither in the CFS, nor in the healthy control group (Figure 1A and 1B). No significant differences in HI antibody titers between both groups were observed prior to vaccination or at day 22. Compared to the healthy controls, CFS patients demonstrated similar seroprotection rates (both pre- and post-vaccination) and seroresponse rates against all three virus-strains (Table 2).

**Table 2:** Humoral and cellular immune responses

	**Chronic fatigue syndrome patients (n=20)**	**Healthy controls (n=20)**	***p*-value**
**Seroprotection rate pre-vaccination (day 1)**			
H3N2	13 (65)	12 (60)	0.744
H1N1	4 (20)	5 (25)	0.705
B	16 (80)	17 (85)	0.677
H3N2, H1N1, and B	2 (10)	4 (20)	0.376
**Seroprotection rate post-vaccination (day 22)**			
H3N2	18 (90)	20 (100)	0.147
H1N1	18 (90)	18 (90)	1.000
B	18 (90)	20 (100)	0.147
H3N2, H1N1, and B	15 (75)	18 (90)	0.212
**Seroresponse rate post-vaccination (day 1 to 22)**			
H3N2	10 (50)	12 (60)	0.525
H1N1	13 (65)	12 (60)	0.744
B	2 (10)	3 (15)	0.633
H3N2, H1N1, and B	1 (5)	2 (10)	0.548
**IL-10 production at baseline (day 1, pg/ml)**			
H3N2	4.5±10.9	2.5±5.0	0.457
H1N1	65.6±127.4	35.3±47.2	0.329
B	19.3±56.0	10.5±20.2	0.514
**Treg at baseline (day 1, %)**			
Unstimulated	2.8±1.2	2.7±1.5	0.733
**IFN-γ production post-vaccination (day 8, pg/ml)**			
H3N2	162.1±220.5	116.0±180.8	0.474
H1N1	578.3±828.8	382.5±446.1	0.360
B	316.4±520.7	418.1±528.9	0.544
**IL-4 production post-vaccination (day 8, pg/ml)**			
H3N2	0.0±0.0	4.3±13.5	0.169
H1N1	0.0±0.0	7.7±20.2	0.105
B	2.9±8.8	0.0±0.0	0.163
**IL-5 production post-vaccination (day 8, pg/ml)**			
H3N2	1.4±4.3	1.7±4.7	0.811
H1N1	1.2±3.4	1.5±4.5	0.788
B	0.8±3.4	3.4±6.4	0.114

Data are presented as absolute numbers with percentages in brackets or as mean ± standard error of the mean.

### Cellular immune response

Prior to vaccination, T cells proliferated upon phytohemagglutinin (PHA) and virus stimulation in both CFS patients and healthy controls (respectively 14,883±4,265counts and 22,997±3,902 counts), indicating that these T cells are functional (Figure 1C and 1D). Cellular proliferation showed no significant differences between CFS patients and healthy controls at baseline (Table 2). Of note, in absolute counts, CFS patients showed at baseline a lower T cell proliferation upon stimulation in comparison with controls. Interleukin-10 (IL-10) production and the number of regulatory T lymphocytes (Treg) were not significantly different between CFS patients and healthy controls at baseline (Table 2).

Vaccination induced a significant increase in cellular proliferation in CFS patients after stimulation with influenza strain H3N2 and B, and a trend in the same direction for strain H1N1, whereas in healthy controls vaccination induced no significant change in cellular proliferation after stimulation with any of the three virus-strains (Figure 1C and 1D and Table 2).

Post-vaccination, T cell proliferation showed no significant differences between CFS patients and controls, neither for the three different virus strains (Figure 1C and 1D), nor for PHA (respectively 34,964±4433 counts and 15,406±2719 counts). Of note, in absolute counts, CFS patients showed post-vaccination more proliferating T cells upon stimulation with all three virus-strains, compared to controls. Interferon gamma (IFN-γ), interleukin-4 (IL-4), and interleukin-5 (IL-5) production post-vaccination were not significantly different between CFS patients and healthy controls (Table 2).

## Discussion

To the best of our knowledge, we are the first to explore both the humoral and cellular immune responses after influenza vaccination in CFS patients. The hypothesized immunological aberrancy claimed to be operative in CFS could not be confirmed by our data.

Both CFS patients and healthy controls showed a significant increase in humoral immune responses from pre- to post-vaccination for virus-strains H3N2 and H1N1. Virus-strain B did not show such an increase from day 1 to day 22, but for this virus-strain the HI titer was already high prior to vaccination.

In a previous study, only the effect of influenza vaccination on the antibody response of CFS patients was determined in 40 CFS patients and 21 matched healthy volunteers [22]. In accordance with our study, influenza vaccination provided protective antibody levels. Our study gives additional information on cellular immune responses in CFS patients.

We observed that, although not statistically significant, the incorporation of tritium thymidine by proliferating T cells at baseline was numerically lower in patients suffering from CFS in comparison to healthy controls. To investigate whether it is worthwhile to investigate this trend in more detail, we studied immunological mechanisms commonly known to suppress immunity. First, the immunosuppressive cytokine IL-10, which is able to inhibit T cell proliferation, was assessed [23]. However, production at baseline of IL-10 was similar in patients and controls. Secondly, Treg are known to negatively modulate T cell responses [24]. Nevertheless, the percentage of Treg in CFS patients at baseline did not differ from the controls. Another possible explanation for the decreased number of proliferating T lymphocytes in CFS patients is elevated numbers of co-inhibitory molecules, like programmed death-1, [25,26] cytotoxic T lymphocyte antigen-4, [25] or B and T lymphocyte attenuator [27]. Whether inhibitory mechanisms play a role in CFS patients need to be explored in more detail.

From pre- to post-vaccination, CFS patients showed a significant increase in cellular proliferation in two out of three virus-strains and a trend in the same direction for the third virus-strain, whereas healthy controls did not show a significant change in proliferation from day 1 to day 8. In absolute counts, CFS patients showed post-vaccination more proliferating T cells for all three virus-strains, compared to controls. This non-significant elevation in cellular immune responses could not be explained by an increase in the levels of cytokines IFN-γ, IL-4, and IL-5, cytokines involved in the type 1 and type 2 helper T cell responses, suggesting a type 0 helper T cell response in CFS patients after vaccination.

### Limitations of the study

Given the exploratory nature of this study, group sizes were relatively small and not based on power calculations. Consequently, subtle differences between CFS patients and healthy controls may have gone unnoticed. However, the sample size of our study was sufficiently high to show that CFS patients are able to mount a significant protective antibody response and a sufficient cellular immune response upon a single shot of influenza vaccine.

## Conclusions

In conclusion, putative aberrations in immune responses in CFS patients were not evident for immunity towards influenza. We show that CFS patients are able to mount a protective antibody response and a sufficient cellular immune response upon a single shot of influenza vaccine, which is comparable with healthy controls. Therefore, standard seasonal influenza vaccination is thus justified and, when indicated, should be recommended for patients suffering from CFS.

## Methods

### Participants

The study population consisted of a group of CFS patients (n=20) and a group of healthy controls (n=20). CFS patients fulfilled the Centre for Disease Control and prevention criteria for CFS [1] and were referred for cognitive behavior therapy to the Expert Centre for Chronic Fatigue of the Radboud University Nijmegen Medical Centre (RUN-MC, Nijmegen, the Netherlands). CFS patients were asked to bring a gender- and age-matched non-fatigued friend as a control. Fatigue severity was measured by the fatigue severity subscale of the Checklist Individual Strength (CIS-fatigue) [28,29]. A cutoff score of ≥35 points on this subscale indicates severe fatigue and a score of <27 points signifies normal fatigue feelings. All participants were between 18 and 60 years old. The local ethics committee of the RUN-MC approved the study and all participants provided written informed consent.

### Vaccination and blood collection

Between September 2010 and January 2011, all participants were intramuscularly vaccinated with a single dose of the inactivated trivalent split influenza vaccine (Vaxigrip^R^, Sanofi Pasteur MSD, Hoofddorp, the Netherlands), which contained inactivated, split virion of the three influenza strains (A/H3N2/Perth/16/2009, A/H1N1/California/7/2009, and B/Brisbane/60/2008).

Peripheral blood mononuclear cells (PBMC) were collected at baseline (day 1) and 7 days after vaccination (day 8), and serum was collected at baseline and 21 days after vaccination (day 22) [30].

### Humoral immune response

The humoral immune responses on influenza vaccination were measured in serum by the HI antibody test as described previously [31]. The virus antibody responses were measured at day 1 and day 22 for the three different influenza strains of the vaccine (A/H3N2/Perth/16/2009, A/H1N1/California/01/2010, and B/Florida/004/2006). Seroprotection was defined as an antibody titer of at least 1:40 [31,32]. Post-vaccination seroresponse was defined as at least a four-fold increase in titers [32].

### Cellular immune response

The cellular immune responses were measured by T lymphocyte proliferation and cytokine secretion of PBMC collected at day 1 and 8, and the presence of Treg at day 1.

For analysis of lymphocyte proliferation and cytokine secretion, 1.5x10^5^ PBMC were added per well of a 96-wells plate in culture medium (RPMI 1640 supplemented with 4% human serum albumin). In the proliferation assay, PBMC were incubated with 1μg/ml PHA and with a 1:10 dilution of the separate virus-strains (A/H3N2/Perth/25/11/2008, A/H1N1/California/01/2010, and B/Florida/05/11/2008). After 48 hours of culture, supernatant was harvested to analyze cytokine production. The Th1/Th2 11plex kit (eBioscience, San Diego, CA) was used according to the manufacturer’s protocol in order to measure IL-10 at day 1 and IFN-γ, IL-4, and IL-5 at day 8. After four days of culture, 1 μCi ^3^[H]-thymidine (MP Biomedicals, Santa Ana, California, USA) was added to each well for overnight incubation to measure T lymphocyte proliferation.

Multi-color flow cytometric analysis was performed on unstimulated PBMC collected at day 1 according to the manufacturer’s protocol. Cells were stained for anti-CD4/FITC (Beckman Coulter), anti-CD25/PE-Cy7 (BD Biosciences, Breda, the Netherlands), anti-CD127/PE (BD Bioscience), and anti-FOXP3/APC (clone PCH101, eBioscience). Treg were defined as CD4+CD25++CD127-FOXP3+ and were expressed as a percentage of CD4+CD25++CD127- cells.

### Statistics

Statistical analyses were performed using PASW for Windows®, version 18.0.2 (Armonk, New York, USA). Independent samples *t* tests were performed to assess differences in numerical variables at day 1, 8, and 22. Paired *t* tests were used to assess changes in numerical variables from pre- to post-vaccination. Chi-square tests were performed to compare groups on categorical variables. Differences were considered statistically significant at *p*<0.05.

## Abbreviations

CFS: Chronic fatigue syndrome; HI: Hemagglutination-inhibition; IFN-γ: Interferon gamma; IL-4: Interleukin-4; IL-5: Interleukin-5; IL-10: Interleukin-10; PBMC: Peripheral blood mononuclear cells; PHA: Phytohemagglutinin; RUN-MC: Radboud University Nijmegen Medical Centre; Treg: Regulatory T lymphocytes.

## Competing interests

The corresponding author and the co-authors have no conflicts of interest to declare.

## Authors’ contributions

HP is primary investigator and is responsible for patient recruitment, data collection, data analysis, and drafting the manuscript. HvL and JdV obtained funding for the study. HvL, JdV, RT, JP, SM, CvH, and HP designed the study. GB and LE assisted in patient recruitment. FS was responsible for the hemagglutination-inhibition antibody test. JP assisted in data collection and data analysis. HvL, JdV, RT, GB, LE, and FS supervised the study. All authors read and approved the final manuscript.

## References

[B1] Fukuda K Straus SE Hickie I Sharpe MC Dobbins JG Komaroff A International Chronic Fatigue Syndrome Study Group The chronic fatigue syndrome: a comprehensive approach to its definition and study Ann Intern Med 1994 121 12 953 959 7978722 10.7326/0003-4819-121-12-199412150-00009

[B2] Lorusso L Mikhaylova SV Capelli E Ferrari D Ngonga GK Ricevuti G Immunological aspects of chronic fatigue syndrome Autoimmun Rev 2009 8 4 287 291 10.1016/j.autrev.2008.08.003 18801465

[B3] Castell BD Kazantzis N Moss-Morris RE Cognitive behavioral therapy and graded exercise for chronic fatigue syndrome: a meta-analysis Clin Psychol: Sci Pract 2011 18 4 311 324 10.1111/j.1468-2850.2011.01262.x

[B4] Chambers D Bagnall AM Hempel S Forbes C Interventions for the treatment, management and rehabilitation of patients with chronic fatigue syndrome/myalgic encephalomyelitis: an updated systematic review J R Soc Med 2006 99 10 506 520 10.1258/jrsm.99.10.506 17021301 PMC1592057

[B5] Malouff JM Thorsteinsson EB Rooke SE Bhullar N Schutte NS Efficacy of cognitive behavioral therapy for chronic fatigue syndrome: a meta-analysis Clin Psychol Rev 2008 28 5 736 745 10.1016/j.cpr.2007.10.004 18060672

[B6] Nijhof SL Bleijenberg G Uiterwaal CS Kimpen JL van de Putte EM Effectiveness of internet-based cognitive behavioural treatment for adolescents with chronic fatigue syndrome (FITNET): a randomised controlled trial Lancet 2012 379 9824 1412 1418 10.1016/S0140-6736(12)60025-7 22385683

[B7] Price JR Mitchell E Tidy E Hunot V Cognitive behaviour therapy for chronic fatigue syndrome in adults Cochrane Database Syst Rev 2008 3 CD001027 18646067 10.1002/14651858.CD001027.pub2 PMC7028002

[B8] Prins JB Bleijenberg G Bazelmans E Elving LD de Boo TM Severens JL van der Wilt GJ Spinhoven P van der Meer JW Cognitive behaviour therapy for chronic fatigue syndrome: a multicentre randomised controlled trial Lancet 2001 357 9259 841 847 10.1016/S0140-6736(00)04198-2 11265953

[B9] Stulemeijer M de Jong LW Fiselier TJ Hoogveld SW Bleijenberg G Cognitive behaviour therapy for adolescents with chronic fatigue syndrome: randomised controlled trial BMJ 2005 330 7481 14 10.1136/bmj.38301.587106.63 15585538 PMC539840

[B10] Whiting P Bagnall AM Sowden AJ Cornell JE Mulrow CD Ramirez G Interventions for the treatment and management of chronic fatigue syndrome: a systematic review JAMA 2001 286 11 1360 1368 10.1001/jama.286.11.1360 11560542

[B11] Brooks JC Roberts N Whitehouse G Majeed T Proton magnetic resonance spectroscopy and morphometry of the hippocampus in chronic fatigue syndrome Br J Radiol 2000 73 875 1206 1208 11144799 10.1259/bjr.73.875.11144799

[B12] Chaudhuri A Condon BR Gow JW Brennan D Hadley DM Proton magnetic resonance spectroscopy of basal ganglia in chronic fatigue syndrome Neuroreport 2003 14 2 225 228 10.1097/00001756-200302100-00013 12598734

[B13] de Lange FP Kalkman JS Bleijenberg G Hagoort P van der Meer JW Toni I Gray matter volume reduction in the chronic fatigue syndrome Neuroimage 2005 26 3 777 781 10.1016/j.neuroimage.2005.02.037 15955487

[B14] de Lange FP Koers A Kalkman JS Bleijenberg G Hagoort P van der Meer JW Toni I Increase in prefrontal cortical volume following cognitive behavioural therapy in patients with chronic fatigue syndrome Brain 2008 131 Pt 8 2172 2180 18587150 10.1093/brain/awn140

[B15] Mathew SJ Mao X Keegan KA Levine SM Smith EL Heier LA Otcheretko V Coplan JD Shungu DC Ventricular cerebrospinal fluid lactate is increased in chronic fatigue syndrome compared with generalized anxiety disorder: an in vivo 3.0 T (1)H MRS imaging study NMR Biomed 2009 22 3 251 258 10.1002/nbm.1315 18942064

[B16] Puri BK Counsell SJ Zaman R Main J Collins AG Hajnal JV Davey NJ Relative increase in choline in the occipital cortex in chronic fatigue syndrome Acta Psychiatr Scand 2002 106 3 224 226 10.1034/j.1600-0447.2002.01300.x 12197861

[B17] Tomoda A Miike T Yamada E Honda H Moroi T Ogawa M Ohtani Y Morishita S Chronic fatigue syndrome in childhood Brain Dev 2000 22 1 60 64 10.1016/S0387-7604(99)00111-4 10761837

[B18] Schillings ML Kalkman JS van der Werf SP van Engelen BG Bleijenberg G Zwarts MJ Diminished central activation during maximal voluntary contraction in chronic fatigue syndrome Clin Neurophysiol 2004 115 11 2518 2524 10.1016/j.clinph.2004.06.007 15465441

[B19] Ortega-Hernandez OD Shoenfeld Y Infection, vaccination, and autoantibodies in chronic fatigue syndrome, cause or coincidence? Ann N Y Acad Sci 2009 1173 600 609 10.1111/j.1749-6632.2009.04799.x 19758205

[B20] Nancy AL Shoenfeld Y Chronic fatigue syndrome with autoantibodies–the result of an augmented adjuvant effect of hepatitis-B vaccine and silicone implant Autoimmun Rev 2008 8 1 52 55 10.1016/j.autrev.2008.07.026 18725327

[B21] Rosenblum H Shoenfeld Y Amital H The common immunogenic etiology of chronic fatigue syndrome: from infections to vaccines via adjuvants to the ASIA syndrome Infect Dis Clin North Am 2011 25 4 851 863 10.1016/j.idc.2011.07.012 22054760

[B22] Sleigh KM Danforth DG Hall RT Fleming JA Stiver HG Double-blind, randomized study of the effects of influenza vaccination on the specific antibody response and clinical course of patients with chronic fatigue syndrome Can J Infect Dis 2000 11 5 267 273 18159300 10.1155/2000/602862 PMC2094778

[B23] Taga K Tosato G IL-10 inhibits human T cell proliferation and IL-2 production J Immunol 1992 148 4 1143 1148 1737931

[B24] Levings MK Sangregorio R Roncarolo MG Human cd25(+)cd4(+) t regulatory cells suppress naive and memory T cell proliferation and can be expanded in vitro without loss of function J Exp Med 2001 193 11 1295 1302 10.1084/jem.193.11.1295 11390436 PMC2193376

[B25] Coyle AJ Gutierrez-Ramos JC The expanding B7 superfamily: increasing complexity in costimulatory signals regulating T cell function Nat Immunol 2001 2 3 203 209 10.1038/85251 11224518

[B26] Keir ME Butte MJ Freeman GJ Sharpe AH PD-1 and its ligands in tolerance and immunity Annu Rev Immunol 2008 26 677 704 10.1146/annurev.immunol.26.021607.090331 18173375 PMC10637733

[B27] Watanabe N Gavrieli M Sedy JR Yang J Fallarino F Loftin SK Hurchla MA Zimmerman N Sim J Zang X BTLA is a lymphocyte inhibitory receptor with similarities to CTLA-4 and PD-1 Nat Immunol 2003 4 7 670 679 10.1038/ni944 12796776

[B28] Dittner AJ Wessely SC Brown RG The assessment of fatigue: a practical guide for clinicians and researchers J Psychosom Res 2004 56 2 157 170 10.1016/S0022-3999(03)00371-4 15016573

[B29] Vercoulen JH Swanink CM Fennis JF Galama JM van der Meer JW Bleijenberg G Dimensional assessment of chronic fatigue syndrome J Psychosom Res 1994 38 5 383 392 10.1016/0022-3999(94)90099-X 7965927

[B30] Mulder SF Jacobs JF Olde Nordkamp MA Galama JM Desar IM Torensma R Teerenstra S Mulders PF Vissers KC Punt CJ Cancer patients treated with sunitinib or sorafenib have sufficient antibody and cellular immune responses to warrant influenza vaccination Clin Cancer Res 2011 17 13 4541 4549 10.1158/1078-0432.CCR-11-0253 21712444

[B31] de Jong JC Palache AM Beyer WE Rimmelzwaan GF Boon AC Osterhaus AD Haemagglutination-inhibiting antibody to influenza virus Dev Biol (Basel) 2003 115 63 73 15088777

[B32] Pollyea DA Brown JM Horning SJ Utility of influenza vaccination for oncology patients J Clin Oncol 2010 28 14 2481 2490 10.1200/JCO.2009.26.6908 20385981

